# Is Striae Gravidarum related to Cesarean Scar and Peritoneal Adhesions?

**DOI:** 10.12669/pjms.343.14288

**Published:** 2018

**Authors:** Esra Yasar Celik, Ali Ozgur Ersoy, Ebru Ersoy, Ozlem Yoruk, Aytekin Tokmak, Yasemin Tasci

**Affiliations:** 1 Esra Yasar Celik, Department of Obstetrics and Gynecology, Zekai Tahir Burak Women’s Health Care Training and Research Hospital, Ankara, Turkey; 2 Ali Ozgur Ersoy, Department of Obstetrics and Gynecology, Zekai Tahir Burak Women’s Health Care Training and Research Hospital, Ankara, Turkey; 3 Ebru Ersoy, Department of Obstetrics and Gynecology, Zekai Tahir Burak Women’s Health Care Training and Research Hospital, Ankara, Turkey; 4 OzlemYoruk, Department of Obstetrics and Gynecology, Zekai Tahir Burak Women’s Health Care Training and Research Hospital, Ankara, Turkey; 5 AytekinTokmak, Department of Obstetrics and Gynecology, Zekai Tahir Burak Women’s Health Care Training and Research Hospital, Ankara, Turkey; 6 YaseminTasci, Department of Obstetrics and Gynecology, Zekai Tahir Burak Women’s Health Care Training and Research Hospital, Ankara, Turkey

**Keywords:** Adhesions, Cesarean section, Keloid, Scar, Striae distensae, Stretchmark, Surgical complication

## Abstract

**Objective:**

To evaluate the relationship between striae gravidarum (SG) score and abdominal scar characteristics together with intraperitoneal adhesion (IPA) grades of patients who were hospitalized for second cesarean delivery.

**Methods:**

A total of 145 consecutive women undergoing scheduled cesarean section (CS) in a tertiary level maternity hospital between November 2013 and January 2014 were included in the study. All women had transverse suprapubic skin incision due to the previous CS and none of them had a history of vaginal delivery. Patients were classified according to the SG status, as women with no SG: Group-1(n=53), mild SG: Group-2(n=27) and severe SG: Group 3(n=65). Groups were compared between themselves with regard to various sociodemographic properties, cesarean scar characteristics and IPA scores.

**Results:**

No significant difference in the length, width and color of the scar was detected among groups. While flat scar was the most prominent form of scar, the elevated scar was significantly more frequent in Group-1 compared to other groups (*p*=0.009). IPA grades were 0 or 1 in 77.3% of Group-1, 81.3% of Group-2 and 76% of Group-3. There was no significant difference in IPA scores between groups (*p*=0.884). After combining CS scar characteristics (flat, depressed and elevated) and SG status [SG (+) or SG (-)], we found no significant difference between the groups in terms of IPA severity.

**Conclusion:**

Striae gravidarum (SG) was found to be associated with scar characteristics, but not associated with the severity of intraperitoneal adhesion (IPA).

## INTRODUCTION

Striae gravidarum (SG, also called’ striae distensae’) is a common condition observed in pregnancy to various extents. Many risk factors have been put forth to be associated with the development to striae, like maternal age, ethnicity, weight, weight gain during pregnancy, birth weight of the newborn, but the exact etiology of striae development is still unclear.^1^-^4^ During pregnancy, fast stretching of the skin leads to cleavage of collagen fibers that may cause easy separation.^5^ In the skin, the amount and type of collagen determines elasticity and striae gravidarum is thought to be the result of poor skin elasticity. Structural changes in connective tissue due to the hormonal effects and reduced elastin and fibril in the dermis cause striae gravidarum formation.^6^

Cesarean section (CS) is one of the most common operation performed worldwide which carries the potential risk of serious complications due to the intraperitoneal adhesions, such as bladder and bowel injury, infertility or chronic pelvic pain.^7^-^9^ It is very difficult to predict the presence of intraperitoneal adhesions (IPA) and whether complications will develop in patients who have undergone previous abdominal surgery. Therefore, it is very important to anticipate adhesions, to take necessary preoperative measures in patients who may have adhesions, and to refer these patients to tertiary centers in order to prevent possible complications. Currently, there is no reliable way to predict the severity and extent of adhesions prior to repeat surgeries.

The protective effect of elastin has been shown in IPA formation and development of SG, and it is believed that SG, abdominal scar and peritoneal adhesion formation share similar pathways of tissue healing.^10^ The aim of this study was to investigate whether SG and abdominal scar characteristics have any predictive value for the severity of IPA in patients who have undergone scheduled second CS.

## METHODS

One hundred forty five consecutive women hospitalized for CS between November 2013- January 2014 were included in the present cross sectional study, each having one previous CS history with low segment transverse skin incision, with no vaginal delivery history. Approval from the Institutional Review Board of the Zekai Tahir Burak Women’s Health Care Training and Research Hospital (Approval Date/Number: 23.08.2013/19) and written informed consent from all participants were obtained before recruiting patients for the study. The research was conducted in accordance with the Helsinki Declaration.

The age, number of gravidity and parity, gestational week at present and previous delivery, period between two deliveries (years), weight gain through out pregnancy (gram) and body mass index (BMI; calculated as weight in kilograms divided by height in meters squared; i.e.kg/m^2^) at hospitalization were noted. Striae score of the abdomen, presence of striae on legs, breasts and buttocks and characteristics of abdominal incision scar were also recorded. Davey scoring system was used to define the severity of SG.^2^ According to this scoring system, the abdomen was divided into quadrants using the midline and horizontal line through the umbilicus.Each quadrant was scored as 0(=clear skin), 1(=moderate number of striae) or 2(=many striae). Total sum of scores ranged from 0 to 8. The severity of striae gravidarum was divided into three categories, 0 (absent), 1 to 2 (mild), and 3 to 8 (severe), as assessed by Davey’s score. All patients were examined by the same investigators (EYC and EE) for the standardization of the scoring system. The length and width of the scar was measured by a ruler. The color of the scar was recorded according to the pigmentation and the level of the scar was defined as depressed, flat or elevated (hypertrophic). Presence of keloid was also recorded.

Data obtained from obstetric history for every woman were as follows, the time, indication and gestational week of the previous CS, history of polyhydramnios, gestational diabetesmellitus (GDM) and fetal macrosomy, weight gain during pregnancy and BMI at delivery, smoking habit, and use of any medication and presence of any chronic illnesses. Exclusion criteria were: endometriosis and pelvic inflammatory disease history, previous abdominal or pelvic surgery other than CS; vaginal delivery history, multiple pregnancy, corticosteroid usage; post operative infectious complications history such as wound infection, endometritis or abscesses which were thought to be risk factors for intraperitoneal adhesions,SG and wound healing respectively.

Intraoperative grading of IPAs was done by the same investigators (EYC and EE) according to the modified Blauer classification which defines intra abdominal adhesions in five categories (0-4).

- Grade 0: Complete absence of adhesions.
- Grade 1, 2: Localised filmy easily separable adhesions.
- Grade 3: Dense, extensive adhesions.
- Grade 4: Adhesions of the intestines to the abdominal wall or to the uterus.

Adhesion of the uterus to the abdominal wall or rectovaginal pouch obliterated by dense adhesions.^11^

All pregnant women recruited for the study were divided into three groups.

***Group-1:*** women with no striae gravidarum in previous and present pregnancy.

***Group-2:*** women with mild striae gravidarum.

***Group-3:*** women with severe striae gravidarum.

Groups were compared via scar characteristics and intra abdominal adhesion grades.

### Statistical Analysis

The Statistical Package for Social Science version15.0 software (SPSS, Chicago, IL., USA) was used to conduct the statistical analyses. Anthropometric and scar features were categorized as categorical variables or continuous variables. Comparisons between the groups were made using the Student’st-test or Mann- Whitney *U* test as appropriate. Categorical variables were compared using the χ^2^ test or Fisher’s exact test as appropriate. One Way Anovaand Kruskal- Wallis variance analysis were used for multi- group comparison of continuous variables. If the differences were significant, pair –wise comparisons would be based on the Mann-Whitney U-test or Bonferroni correction to establish which subgroups were different. All of the reported p-values were two-tailed, and those less than 0.05 were considered to be statistically significant. The statistical program on the website of the statistical department of the University of British Colombia was used to calculate the sample size and the power of our study (https://www.stat.ubc.ca/~rollin/stats/ssize/b1.html). According to SG status, the inclusion of 45 patients in each study group with 80% confidence interval and p <0.05 significance level was calculated as sufficient for the sample size of our study.

## RESULTS

Of 145 women recruited in the study, 53 women had no SG (Group-1), 27 had mild (Group-2) and 65 had severe SG (Group-3). Some of the sociodemographic characteristics of the patients and data obtained from their obstetric histories are summarized in Table-I. The median age was 31 (27-34) in Group-1, 27 (25-29) in Group-2 and 28 (24-30) in Group-3 and the difference among them was significant (*p*=0.001). Median BMI at the time of the previous CS was 27(25-29) in Group-1, 29 (24-31) in Group-2 and 30 (26-33) in Group-3 and the difference among them was significant (*p=*0.003). BMI at the time of the present CS was 27 (26-30) in Group-1, 29 (27-31) in Group-2 and 32 (30-35) in Group-3, this difference between the groups was also significant (*p<*0.001). However, weight gain during pregnancy was not different among groups in both previous and present pregnancies (p>0.05) (Table-I). SG history in the first pregnancy and presence of striae on other body parts (breasts, buttocks and legs) were increasing with the increasing severity of SG and the difference among groups was statistically significant for every clinical entity (Table-I). Family history of SG was significantly more prevalent in Group-3. There was no significant difference in level of income, education and indication of the first CS among groups. Also, three groups were similar regarding the risk factors of SG; the incidence of smoking habit GDM, polyhydramnios, and birth weight of the newborn (Table-I).

**Table-I:** Some characteristics of the groups according to Davey scoring system.

Characteristics	Group-1 (n = 53) No SG	Group-2 (n = 27) Mild SG	Group-3 (n = 65) Severe SG	*p*-value
Age (years)	31 (27-34)	27 (25-29)	28 (24-30)	0.001
Gravidity (2 / 3 / 4 / >4)	40/10/3/0	20/6/1/0	53/11/1/0	0.826
Abortus (0 / 1 / 2)	39/11/3	20/6/1/0	51/13/1/0	0.952
Education (≤ 5 years / middle / high / university)	14/15/11/13	6/6/9/6	17/20/12/6	0.433
Level of income(low / middle / high)	17/21/15	4/17/6	18/33/14	0.322
Smoking habit (-/+)	49/4	25/2	54/11	0.215
SG in first pregnancy (-/+)	52/1	6/21	3/62	<0.001
Family history of SG (-/+)	27/26	16/11	21/44	0.027
Striae on buttocks (-/+)	3/50	4/23	18/47	0.007
Striae on leg (-/+)	8/45	8/19	31/34	0.001
Striae on breasts (-/+)	0/53	0/27	6/59	0.021
Period between two C/S (years)	4.99±2.87	4.09±1.91	4.82±2.90	0.516
GW at first C/S	39 (38-40)	40 (38-40)	40 (39-41)	0.039
Birth weight of first baby (gram)	3309±581	3281±587	3327±586	0.884
BMI at previous C/S (kg/m^2^)	27 (25-29)	29 (24-31)	30 (26-33)	0.003
BMI at present C/S (kg/m^2^)	27 (26-30)	29 (27-31)	32 (30-35)	<0.001
GW at present C/S	38.42±1.08	38.63±0.88	38.74±0.67	0.335
Birth weight of second baby (gram)	3315±297	3270±458	3351±381	0.658

SG: Striae gravidarum; GW: Gestational week; BMI: Body mass index; C/S: Cesarean section.

Data are expressed as the mean±SD, median (25th-75th interquartile range) or number of cases as appropriate.’(-/+)’ refers to number of the ’absence or presence’ of the mentioned characteristic.

P-values were calculated using Student’s t-test, Mann Whitney U test, Chi Square test,

One Way Anova and Kruskal-Wallis variance analysisas appropriate.

There was no significant difference in the length, width and color of the scar among groups (Table-II). Although flat scar was the most prominent scar form among all groups, the level of scars were significantly different among groups (p=0.009). No difference was detected in the presence of keloid among groups (p=0.325). IPA grade was 0 or1 in 77.3% of Group-1, 81.3% of Group-2 and 76% of Group-3. There was no significant difference in IPA scores among groups (*p*=0.884). We compared IPA (-) (Grade 0) and IPA (+) (Grade1-4) patients, after combining CS scar properties (flat, depressed and elevated) with SG status [SG (+) or SG(-) ] and we found no difference between IPA groups (Table-III).

**Table-II:** Intraperitoneal adhesion scores and some scar characteristics of the patients according to Davey scoring system.

Characteristics	Group-1 (n=53) No SG	Group-2 (n=27) Mild SG	Group-3 (n=65) Severe SG	*P*-value
Intraperitoneal adhesions Grade 0- 1 / Grade 2-4	41/12	22/5	50/15	0.884
Keloid (-/+)	39/14	22/5	55/10	0.325
Pigmentation (pigmented/non pigmented)	15/38	10/17	25/40	0.489
Scar appearance (flat/depressed/elevated)	39/3/11	20/0/7	53/9/3	0.009
Scar length (cm)	15±1.9	15.11±1.72	15.49±1.98	0.181
Scar width (cm)	2.38±1.79	2.33±1.59	2.26±1.69	0.951

Data are expressed as the mean±SD o rnumber of cases as appropriate.

P-values were calculated using Chi square,

One Way Anova and Kruskal-Wallisvariance analysis as appropriate.

**Table-III:** Comparison of intraperitoneal adhesion groups with the scar appearance and SG status.

Characteristics	IPA Present (n=70)	IPA Absent (n=75)	*p*-value
SG Present+Flat	35 (50%)	39 (52%)	0.81
SG Absent+Flat	20 (28.5%)	18 (24%)	0.53
SG Present+Depressed	4 (5.7%)	5 (6.6%)	1
SG Absent+Depressed	2 (2.8%)	3 (4%)	1
SG Present+Elevated	4 (5.7%)	6 (8%)	0.74
SG Absent+Elevated	5 (7.1%)	4 (5.3%)	0.84

IPA: Intra peritoneal adhesion, SG: Striae gravidarum. Data are expressed as number of cases.

p-values were calculated using Pearson Chi Square or Fisher’s exact test as appropriate.

## DISCUSSION

The primary cesarean delivery rate increased in the last decade and vaginal birth after cesarean delivery declined remarkably.^12^ With large discrepancies among and within different countries, the average global CS rate is approximately 15%.^13^ Adhesion formation, as a complication of previous CS deliveries, may lead to bowel and bladder injury, chronic pelvic pain, infertility, bowel obstruction together with increased operation time and costs.^14^ There is still no reliable way to predict the severity and location of these adhesions before surgery.

Relaxin is a hormone that has been shown to inhibit excessive connective tissue build-up by decreasing collagen production and enhancing collagen breakdown.^15^ Lurie et al.^16^ found that pregnant women with SG had lower serum relaxin levels compared to those without SG. They suggested that connective tissue including higher relaxin levels would be more laxthanones including lower relaxin levels. Data shows that accelerated physical stretching of the skin during pregnancy leads to structural disruption of the elastic fiber network of the skin.^17^ If connective tissue is more thight the at the time of the stretch, there would be more tendencies towards to formation of collagen break down leading to more prominent SG. Brecht et al.^18^ showed that relaxin, a potent vasodilatory and anti fibrotic agent, inhibited early steps in vascular inflammation. These results forced us to establish the hypothesis of that women having higher SG scores are more likely to have more severe IPAs, non- flat scar appearance or keloid. To the best of our knowledge, the present study is the first one investigating the association among SG, IPA and scar characteristics, by means of excluding almost all risk factors for SG and IPA formation. However, the results revealed no association between SG and IPA.

Cakir Gungor et al.^19^ demonstrated that presence of SG had a sensitivity of 95.2% and a specificity of 29.4% for the prediction of IPAs, while having severe SG was less sensitive (80.95%) but more specific (50%) in the prediction of IPAs. In that series of 55 cases, IPA was found to be significantly more prevalent in women with higher SG scores. On the other hand, factors that might affect SG and IPA formation, like parity or number of previous CS was not mentioned in that study. Among scar characteristics only scar appearance (defined as flat, depressed and elevated) was found to be significantly different among groups in the present study.

Hypertrophic scar formation is thought to originate from higher production of collagen, through over expression of transforming growth factor- βeta (TGF-β).^20^ Since reduced elastin in dermal skin is thought to be the causative factor for SG, increased elevated scar forming women with no SG may be related with higher TGF-β which is a potent elastogenic mediator.^21^

Many authors investigated the association between abdominal scar characteristics and IPA, and documented conflicting results. Dogan et al.^22^ reported that 60% of women with hypertrophic scars had some form of adhesions, half of which were dense. In contrast, Salim et al.^20^ and Kahyaoglu et al.^23^ found that depressed scars are significantly associated with increased incidence and severity of adhesions. However, our results do no support those suggestions. When the presence of striae gravidarum which was combined with scar features was assessed, no significant difference was observed between the rates of women with and without IPA in this study.

### Limitation of the study

The lack of previous surgery notes of the some patients was the main limitation of this study. For this reason, surgical procedures performed in the previous CS such as closing or not closing the visceral/parietal peritone, extension of uterine incision, the type and amount of suture material used, and presence of chorioamnionitis were not clear. Although the patients with a history of pelvic inflammatory disease and endometriosis were excluded from the study, these conditions may have led to IPA in silent and/or undiagnosed patients.

## CONCLUSION

SG was found to be associated with scar characteristics, but not associated with IPAs. Further studies with larger sample sizes combining various skin and scar properties may be helpful for prediction of postoperative intra abdominal adhesions.

### Authors’ Contribution

**EYC:** Conceived, designed, did data collection, and writing first draft of manuscript.

**AOE** Did statistical analysis and manuscript writing.

**EE:** Designed and did data collection.

**OY:** Did data collection.

**AT:** Did statistical analysis and editing of manuscript.

**YT:** Conceived and revised manuscript critically.

All authors approved the final version of the manuscript and agree to be accountable for all aspects of this work.
